# Kupffer cells in hepatotoxicity

**DOI:** 10.17179/excli2020-2746

**Published:** 2020-08-14

**Authors:** Reham Hassan

**Affiliations:** 1 Department of Forensic Medicine and Toxicology, Faculty of Veterinary Medicine, South Valley University, Qena, Egypt

## ⁯⁯

***Dear Editor,***

Recently, Gao and colleagues published a study about the role of Kupffer cells in hepatotoxicity (Gao et al., 2020[1]). The authors used a damage model with combined flucloxacillin and CpG-oligodeoxynucleotides in mice. Flucloxacillin is used for the treatment of Gram-negative infections and is known to induce liver damage in a small fraction of patients (Russmann et al., 2005[10]). To recapitulate flucloxacillin induced hepatotoxicity in mice, the antibiotic has been co-administered with CpG-oligodeoxynucleotides, because the latter initiate innate immune responses (Takeshita et al., 2001[13]; Hemmi et al., 2000[3]; Gao et al., 2020[1]). The combination flucloxacillin plus CpG-oligodeoxynucleotides mediates Fas ligand dependent apoptosis of hepatocytes via natural killer cells (Gao et al., 2020[1]; Song et al., 2019[12]). 

In their present study, Gao et al. removed Kupffer cells by treatment of the mice with GdCl_3_ (Gao et al., 2020[1]). Interestingly, the authors observed that GdCl_3_ treated mice showed less liver damage compared to mice that received flucloxacillin plus CpG-oligodeoxynucleotides only. This result suggests that Kupffer cells activate natural killer cells that subsequently induce apoptosis of hepatocytes in this mouse model. 

Kupffer cells are known as important modifiers of hepatotoxicity (Kessler et al., 2014[5]; Reif et al., 2017[9]; Pfeiffer et al., 2015[8]). They filter bacterial fragments and particles out of the sinusoidal blood (Godoy et al., 2013[2]; Köppert et al., 2018[6]) but by the release of cytokines may also contribute to the aggravation of liver damage (Tsutsui and Nishiguchi, 2014[14]; Hou et al., 2017[4]; Leist et al., 2017[7]; Schenk et al., 2017[11]). The present study of Gao and colleagues contributes an important piece of information how hepatotoxic compounds and modifiers of immune cell functions may interact to cause liver damage. 

## Conflict of interest

The authors declare no conflict of interest.
